# Copper Sulfate Supplementation Alleviates Molybdenosis in the Tibetan Gazelles in the Qinghai Lake Basin

**DOI:** 10.3390/toxics12080546

**Published:** 2024-07-27

**Authors:** Guangyang Liu, Xiaoyun Shen

**Affiliations:** 1 School of Life Science and Engineering, Southwest University of Science and Technology, Mianyang 621000, China; guangyangliu@mails.swust.edu.cn; 2 School of Life Sciences, Liaocheng University, Liaocheng 252000, China

**Keywords:** *P. picticaudata*, near threatened, trace element, the animal rescue center, antioxidant effect

## Abstract

Molybdenum (Mo), an essential mineral, plays a key role in the vital activity of the organism. However, excess Mo in the forage will cause loss of appetite, diarrhea, emaciation, bone injury, joint abnormalities, and anemia in animals. In order to study molybdenosis in the *Procapra picticaudata* in the animal rescue center, samples of soils, forages, blood, and liver were collected. The mineral contents of all samples were determined, and the blood parameters were also measured. The results showed that the Mo level in the soil and forage in the animal rescue center was significantly higher than that in healthy pastures (*p* < 0.01). The Mo concentrations in the blood and liver in the *P. picticaudata* from the animal rescue center were also noticeably higher than those in healthy animals (*p* < 0.01). The level of Cu in the blood and liver were noticeably lower than those in healthy *P. picticaudata* (*p* < 0.01). The superoxide dismutase (SOD), glutathione peroxidase (GSH-Px), total antioxidant capacity (T-AOC), and catalase (CAT) were significantly lower than those in healthy animals. Supplementing copper sulfate (CuSO_4_) could significantly decrease the Mo content in the blood, and cure molybdenosis. In summary, the excessive Mo content in the soil and forage in the animal rescue center had greatly affected physiological parameters and antioxidant capacity. It is likely that the molybdenosis of the *P. picticaudata* is caused by the high Mo contents in soils and forages. CuSO_4_ may alleviate molybdenosis in *P. picticaudata*.

## 1. Introduction

The Tibetan gazelle (*Procapra picticaudata*) is an ungulate endemic in China, and classified as near threatened (NT) by the International Union for Conservation of Nature Species Survival Commission [1]. The *P. picticaudata* is a typical alpine and cold desert animal, living in alpine meadows, subalpine steppe meadows, and alpine desert areas between 300 and 5750 m above sea level. They are distributed formerly in Tibet, Qinghai, Gansu, Sichuan, and Xinjiang in Western China [2]. Currently, due to the environmental changes, illegal poaching, and economic development, the living space of the *P. picticaudata* has been greatly compressed, and their behavior of balancing mineral nutrition has been restricted, resulting in an increasing number of *P. picticaudata* in the Qinghai Lake Basin suffering from molybdenosis, represented by diarrhea. After various mineral tests before and after, the soil in some areas of the Qinghai Lake basin was found to contain high levels of molybdenum (Mo), and the excess molybdenum entered the forage and the gazelle’s bodies through the soil–forage–animal cycle; according to the investigation and research, there was also a molybdenosis phenomenon of Przewalski’s gazelle in the same area [3]. However, the causes of high molybdenum levels in soil are not clear and more research is needed. As a result, both nature reserves and animal rescue centers have been set up to protect species and conduct research [4,5]. Animals with molybdenum disease are protected in animal rescue centers [6,7]. Molybdenum is a silver-white metal that is an essential trace element for the human body, animals and plants, as molybdenum is a component of xanthine oxidase, dehydrogenase, aldehyde oxidase, and sulfite oxidase [8,9]. In areas rich in Mo, animals are prone to Mo poisoning, which is caused by excessive Mo content in the soil, drinking water, and forages [10,11]. Ruminants are more sensitive to molybdenum than monogastric animals; its clinical manifestations are continuous diarrhea and coat discoloration, and further manifested as immune function, reproductive function, urinary function, and other disorders [12]. In addition, studies have shown that animal molybdenosis caused by high molybdenum is also manifested in obvious deficiency symptoms of copper (Cu) [13,14]. The metabolism of Cu and Mo and their relationships are very complicated. It has been mentioned in the literature that molybdate and sulfide react with each other to form polythioate in rumen, and Cu reacts with polythioate in rumen to form an insoluble complex which is difficult to be absorbed. In addition, after the thiomolybdate combines with Cu to form chelates, the bioavailability is lost, resulting in a deficiency of Cu. Molybdenosis is generally treated by feeding appropriate amounts of copper sulphate (CuSO_4_) or sodium sulphate (Na_2_SO_4_), as shown in previous studies [15,16,17].

The purposes of this study were to investigate the effect of excessive Mo on the *P. picticaudata* in animal rescue centers of the Qinghai Lake Nature Reserve, and to explore the treatment effect of CuSO_4_ on Mo molybdenosis in the *P. picticaudata*.

## 2. Materials and Methods

### 2.1. Study Pasture

**Figure 1:**
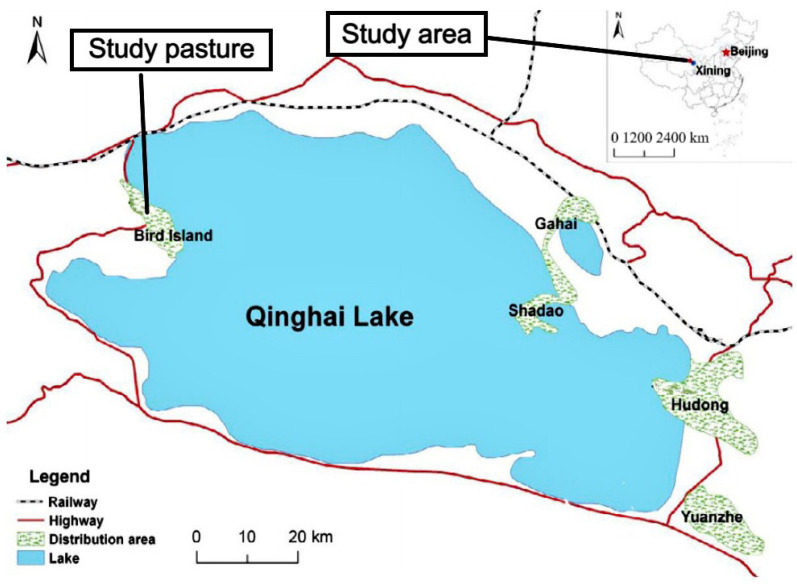
Study pasture.

The ranches of the animal rescue center are located in the Qinghai Lake basin (36°53′12″ N, 96°49′42″ E) (Figure 1), with a plateau continental climate, sufficient light, and strong sunshine. There are vast natural pastures on the shore of the Qinghai Lake, large tracts of fertile farmland and rich mineral resources. The main forage types are *Neotrinia splendens*, *Iris lacteal*, *Stellera chamaejasme*, *Stipa glareosa*, *Stipa penicillata*, and *Artmisia desertorum*, and the main nutritional components of mixed forage in experimental areas are shown in Table 1. It is snowy in winter, rainy in summer and autumn, with sufficient water and abundant rainfall, and has good conditions for the development of animal husbandry. As early as Ancient China, it was an important producing area for horses, cattle, sheep, and other livestock. 

### 2.2. Experimental Design

This experiment was conducted at Qinghai Lake Basin in July–August 2023. First, before the start of the experiment, 20 test *P. picticaudata* were selected, including 10 *P. picticaudata* (1.5 years old, male, 41.33 ± 0.58 kg, severe diarrhea as evidenced by frequent watery stools) affected by molybdenum poisoning from an animal rescue center and 10 healthy *P. picticaudata* (1.5 years old, male, 44.24 ± 0.62 kg) from a healthy pasture, and the mineral content of the soil, pasture, blood and liver of the animals, as well as the hematological indices of the animal’s blood, were measured in the two pastures. 

A further 20 affected *P. picticaudata* (1.5 years old, male, 42.11 ± 0.52 kg, severe diarrhea) were selected from ranches of the animal rescue center. They were randomly divided into two groups with ten animals in each group. Ten *P. picticaudata* were orally treated with CuSO_4_ (Juhengda, Tianjin, China) (100 mg Cd·kg^−1^·body weight) once every 5 days; the other ten *P. picticaudata* were grazing on the pastures without any treatment in the animal rescue center. The clinical status of the animals was observed daily and the elemental content of their blood was determined once every 10 days; hematological indices that were significantly different at the start of the experiment were measured at day 30 after starting treatment. The clinical signs were recorded by direct observation while following the herd on the ranches, noting, in particular, any manifestation of animals affected by the syndrome. The protocol for the present experiment was approved by the Institutional Animal Care and Use Committee of Southwest University of Science and Technology (SWUST2023018, 15 March 2023).

### 2.3. Sample Collection

A total of 20 soil and 20 pasture samples were collected from the affected area. The same amount of soil and pasture samples were taken from the healthy pasture with balanced mineral nutrients. Then, the samples were dried in a drying oven at 40 °C for 72 h to a constant weight. 

The whole blood and serum samples were taken from a jugular vein with a vacuum blood collection vessel containing EDTA-K_2_ (Kangweishi, Hebei, China) anticoagulant or separation gel, respectively. The blood samples were stored at 4 °C for analysis. The serum samples were centrifuged (Thermo Scientific Medifuge, Thermo Fisher Scientific, Dreieich, Germany, centrifugal conditions: 1354× *g* for 10 min), then were stored at −80 °C for further analysis [18].

Liver samples were obtained by liver puncture biopsy. From each group of *P. picticaudata*, 20–25 mg of liver tissue was taken separately from 10 liver samples from each group, making a total of 20 samples; the puncture sites were located by ultrasound. The hair on the right side of the *P. picticaudata* liver site was removed and then the exposed skin was disinfected with alcohol; vitamin K (10 mg·mL^−1^) was administered by intramuscular injection at a dose of 1 mL/goat and the animal was then anesthetized with a local anesthetic, usually 3 mL·kg^−1^ (1% lidocaine). A small incision was made on the right side of the abdomen and a biopsy needle was inserted into the abdominal cavity. While holding their breath, the needle was inserted into the liver and the tissue sample was quickly removed before a bandage was placed over the incision. The collected liver samples were returned to the laboratory within 8 h at a low temperature (4–8 °C) for further processing and analysis.

### 2.4. Determination of Samples

The samples of soils, forages, blood, and liver were digested by the microwave radiation method. During the test, a small amount of distilled water was used to rinse the wall of the PTFE digestion tubes, the samples were weighed (accurately to 0.1 mg) and placed in the digestion tube, 6 mL of nitric acid (HNO_3_) and 1 mL of hydrogen peroxide (H_2_O_2_) were added, shaken well, and left to stand for about 5 min to allow the violent chemical reaction to subside, then the upper lid was tightened. The support for the digestion tube was fitted and the tube was placed on the turntable of the microwave oven. It was confirmed that the pressure sensor and temperature sensor of the digestion instrument were working normally, and then the solution was automatically dissolved according to the microwave dissolution procedure. After the dissolution procedure was completed, the solution was cooled to room temperature, then the cap was removed and the solution was transferred to a PTFE crucible. The dissolution vessel was rinsed with nitric acid and deionized water, then poured into the crucible and after taking up the acid, transferred to a 50 mL volumetric flask, diluted to the scale and then all samples were labelled [18]. 

Finally, the contents of Cu and Mo in the samples were determined by atomic absorption spectrometry (AA-7000, Shimadzu, Kyoto, Japan) and Intelligent Automatic Microwave Dissolver (Touchwin 4.0, Oppler, Shanghai, China) [19,20]. The determination of hemoglobin (Hb), red cell volume (PCV), red blood cells (RBCs), and white cells (WBCs) were determined using a fully automated hematology analyzer (ST-4000, Sysmex-Toa Medical Electronics, Kobe, Japan). The levels of mean erythrocyte hemoglobin volume (MCH), mean hemoglobin concentration (MCHC), and mean erythrocyte volume (MCV) were calculated by the values of Hb, PCV and RBCs. A fully automated biochemical analyzer (OlympusAU640, Olympus Optical Co., Tokyo, Japan) was used to determine the levels of ceruloplasmin (Cp), albumin (ALB), alanine aminotransferase (ALT), alkaline phosphatase (ALP), aspartate aminotransferase (AST), catalase (CAT), cholesterol (Chol), phosphocreatine kinase (CPK), creatinine (CR), globulin (GLB), glutathione peroxidase (GSH-Px), lactate dehydrogenase (LDH), malondialdehyde (MDA), superoxide dismutase (SOD), total protein (TP), and total antioxidant capacity (T-AOC) with commercial test kits (Nanjing Jiancheng Bio-Engineering Institute, Nanjing, China).

### 2.5. Statistical Analyses

Data were analyzed by the statistical package (SPSS, version 23.0, Inc., Chicago, IL, USA) and Data Processing System (DPS, version 19.05, Hangzhou, China) software [21], and were presented in the form of mean ± standard deviation (SD). Differences among the groups were assessed by Student’s *t*-test and a probability level of *p* < 0.01 indicated a significant difference.

## 3. Results

### 3.1. The Mineral Concentrations in Each Group

**Figure 2:**
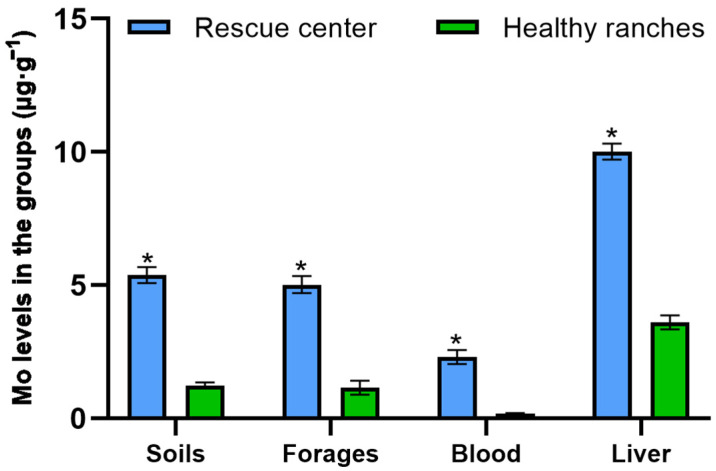
The Mo content in each group. * Indicates significant difference at *p* < 0.01.

**Figure 3:**
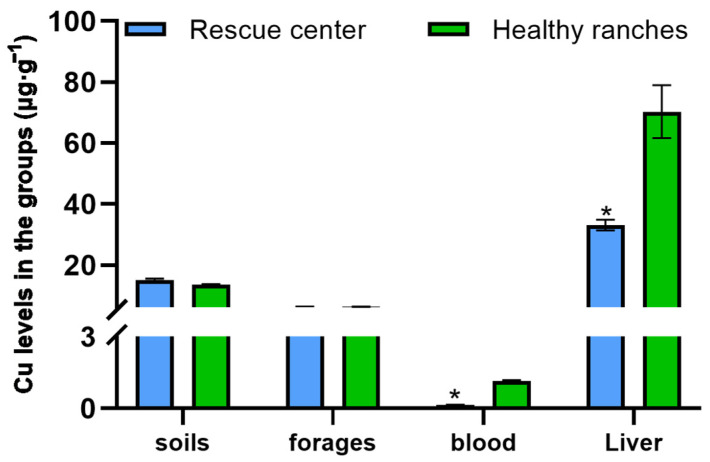
The Cu content in each group. * Indicates significant difference at *p* < 0.01.

The mineral contents in the soil, forage, blood and liver of *P. picticaudata* were shown in Figure 2 and Figure 3. The Mo concentrations in the soil, forage, blood and liver of *P. picticaudata* from the ranches of the rescue center were 5.38 ± 0.27 μg·g^−1^, 4.99 ± 0.18 μg·g^−1^, 2.31 ± 0.09 μg·g^−1^, and 10.02 ± 2.08 μg·g^−1^, respectively, which was noticeably higher than that in the healthy ranches (*p* < 0.01). The Cu concentrations in the blood and liver of *P. picticaudata* were 0.14 ± 0.02 μg·g^−1^ and 33.12 ± 4.89 μg·g^−1^, respectively, which was significantly lower than that of healthy ranches (*p* < 0.01). The Cu content of soils and forages was within the healthy range.

### 3.2. The Physiological Parameters in the Blood of the P. picticaudata

The levels of Hb, MCH, MCV, and PCV in the blood of the *P. picticaudata* from the animal rescue center were noticeably lower than those in healthy *P. picticaudata* (*p* < 0.01, Table 2), while MCHC, RBCs, and WBCs were not affected.

### 3.3. The Biochemical Parameters in the Blood of the P. picticaudata

The levels of ALP, AST, CPK, LDH, and MDA in the *P. picticaudata* from the animal rescue center were noticeably higher than those in healthy *P. picticaudata* (*p* < 0.01, Table 3). The levels of Cp, ALB, GLB, TP, GSH-Px, SOD, CAT, and T-AOC of the *P. picticaudata* from the animal rescue center were significantly lower than those in healthy *P. picticaudata* (*p* < 0.01). There was no significant difference in the ALT, CR, and Chol levels.

### 3.4. Effects of Replenishing CuSO_4_ on the P. picticaudata 

**Figure 4:**
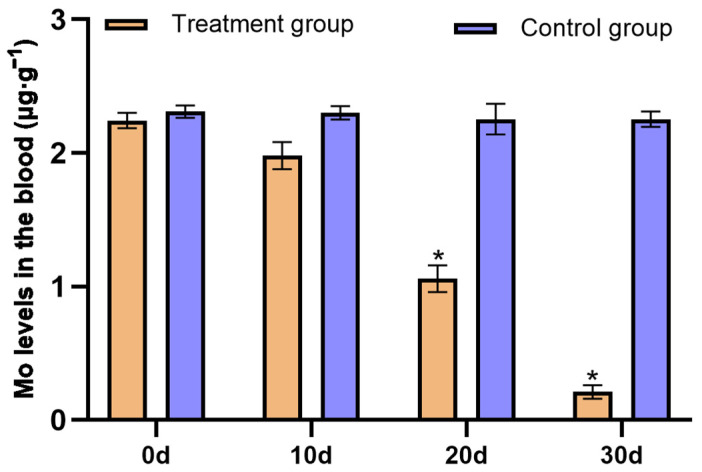
Effect of supplying CuSO_4_ on the Mo content in affected blood. * Indicates significant difference at *p* < 0.01.

**Figure 5:**
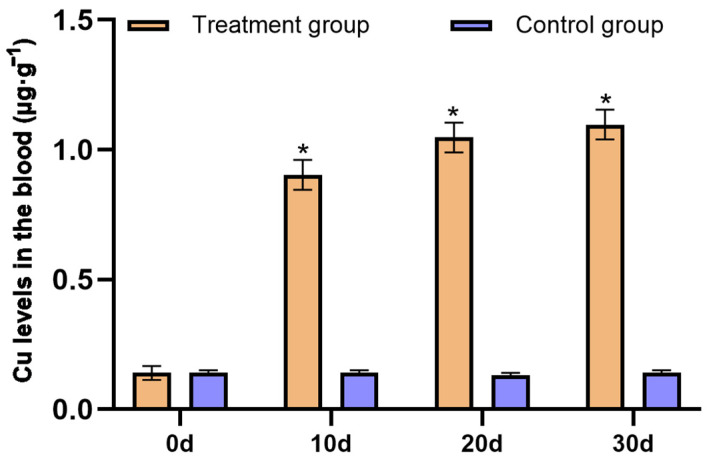
Effect of supplying CuSO_4_ on the Cu content in affected blood. * Indicates significant difference at *p* < 0.01.

Diarrheal symptoms improved or disappeared considerably in all *P. picticaudata* supplemented with CuSO_4_ and did not improve in *P. picticaudata* not supplemented with CuSO_4_. The Mo concentration in the blood of the *P. picticaudata* replenished with CuSO_4_ was gradually decreased and reached a healthy level at day 30, and the concentrations of Cu gradually increased and reached a healthy level at day 30 (Figure 4 and Figure 5). The hematological indices of the *P. picticaudata* fed CuSO_4_ also improved at day 30 and were significantly different from those of the *P. picticaudata* not fed CuSO_4_ (*p* < 0.01, Table 4 and Table 5). This might indicate that molybdenosis *P. picticaudata* could be cured by feeding CuSO_4_.

## 4. Discussion

### 4.1. Effects on P. picticaudata in a High Molybdenum Environment

Mo, a trace element, provides essential nutrition for the normal growth and development of the organism, and improves their production performance [22]. Mo poisoning of ruminants often occurs in such pastures where Mo is naturally enriched or contaminated by industry [23]. In the present study, the Mo level in the soil and forage in the animal rescue center was significantly higher than that in healthy ranches. The Mo content in animal tissues is often influenced by presence status, biological activity, and chymotryptic factors in the soil. Especially, the Mo level in animal blood is quite sensitive to Mo intake. The concentration of Mo in animal blood can rapidly increase with increasing intake. Mo entering the blood becomes a highly dialyzed cation that participates in blood circulation and enters tissues such as bones, muscles, and furs [24,25,26]. Due to the obvious interactions between Mo and Cu, excessive intake of Mo can reduce the Cu utilization in animals, resulting in secondary Cu deficiency [27,28,29]. The analysis of mineral concentrations revealed that the Mo levels in the blood and liver of the *P. picticaudata* in the animal rescue center were 2.31 μg·g^−1^ and 10.02 ± 2.08 μg·g^−1^, which were noticeably higher than that in healthy *P. picticaudata*. The Cu levels in the blood and liver were 0.14 μg·g^−1^ and 33.12 ± 4.89 μg·g^−1^, which were significantly lower than that in healthy *P. picticaudata*. After the *P. picticaudata* of molybdenosis was fed with CuSO_4_, molybdenosis symptoms improved or disappeared considerably, and the concentrations of both Mo and Cu in the blood reached healthy levels at day 30. This study showed that the symptoms of diarrhea, emaciation, joint abnormalities, and lower appetite in the *P. picticaudata* in the animal rescue center can be attributed to molybdenosis, and feeding a certain amount of CuSO_4_ could prevent or cure molybdenosis [30,31]. 

The test of blood parameters found that the values of Hb, MCH, MCV, and PCV in the *P. picticaudata* from the animal rescue center were noticeably lower than those in healthy *P. picticaudata*, implying that molybdenum-poisoned *P. picticaudata* were at risk of anemia. The activity of ALT, AST, and ALP in the serum can reflect the degree of hepatocyte injuries; AST is involved in the process of protein synthesis within the liver and helps maintain normal cell structure and function. When the liver is damaged or diseased, liver cells release more ALP and AST into the bloodstream, so elevated ALP and AST levels may indicate liver problems [32]. In this experiment, the activities of AST and ALP in *P. picticaudata* in the animal rescue center were increased, indicating that high Mo content in the forage led to liver damage. LDH and CPK activities are often used as indicators of muscle and kidney damage. Both LDH and CPK are involved in the process of energy metabolism within muscle cells and kidney cells. LDH promotes the production and conversion of lactic acid, whereas CPK is involved in the process of storing and releasing energy within muscle cells and kidney cells; when muscles and kidneys are damaged or affected by disease, LDH and CPK may be released from damaged muscle cells and kidney cells into the bloodstream, resulting in elevated blood levels of LDH and CPK [33]. The LDH and CPK activities were significantly elevated in the *P. picticaudata* at the animal rescue center, suggesting damage to their kidneys and muscles. ALB is a composite indicator of impaired liver function, and a decrease in the value of this indicator leads to a decrease in albumin production, which is parallel to severe body loss [34,35]. GLB is synthesized by immune organs, and in the presence of antigens like viruses, its synthesis increases [36]. The concentrations of ALB and GLB work together to maintain a constant level of total protein [37]. The reduced ALB and GLB levels in the *P. picticaudata* at the animal rescue center indicated Mo toxicity, causing liver damage and impaired immune system function. Animal diseases are closely linked to free radicals, as disordered metabolism of these radicals can result in reduced immunity [38]. The animal organism can maintain the balance between oxidation and anti-oxidation by utilizing GSH-Px, SOD, and CAT to scavenge excessive free radicals within the organism [39,40]. Cp is a protein containing Cu; it has antioxidant properties that can help scavenge free radicals and harmful oxidants from the body, protecting cells from oxidative damage. GSH-Px is an important enzyme that neutralizes harmful peroxides, protects cells from oxidative stress and maintains intracellular redox homeostasis by reacting reduced glutathione (GSH) with peroxides and converting them to relatively stable water or alcohols [41]. CAT is an important antioxidant enzyme whose main role is to convert hydrogen peroxide produced in the cell into water and oxygen, thus acting as a scavenger of harmful reactive oxygen species. Hydrogen peroxide is a more active oxidizing substance which, if not removed in time, can cause damage to cell structure and function [42]. SOD, a crucial enzyme found abundantly in aerobic organisms, plays a vital role in enhancing the antioxidant capacity of organisms. SOD facilitates a rapid disproportionation reaction of the superoxide anion (O^2−^) to eliminate it, thereby safeguarding cells from damages caused by free radicals [43]. T-AOC is a comprehensive indicator of antioxidant function, showing the body’s resistance to external stimuli and the metabolism of free radicals. MDA, a product of lipid peroxidation, serves as an indicator of the organism’s level of lipid peroxidation [44,45,46,47,48,49]. In this study, enzyme activities and bioprotein index related to antioxidant functions were significantly increased or decreased, indicating an imbalance in the oxidative and antioxidant systems of the *P. picticaudata*.

### 4.2. Supplying CuSO_4_ to Affected P. picticaudata

Molybdenosis mainly occurs in the high molybdenum area of grazing animals; once the animal feed is high in molybdenum forage, it will cause its own molybdenosis, which will appear as diarrhea, hair discoloration, skin reddening and other symptoms, and in severe cases, it will even cause death, so it is very necessary for animals with molybdenosis to take therapeutic measures. It is pointed out in the literature that the oral administration of CuSO_4_ can treat molybdenosis in livestock, the dosage for adult sheep is 1 g d^−1^, and the course of treatment should last for 4 consecutive days. In the rumen of ruminants, Mo will form sulfur molybdate with S. In the intestine, sulfur molybdate will further form a Cu-S molybdate complex with Cu, which will reduce the bioavailability of Mo, interfere with the intestinal absorption of Mo, accelerate the discharge of Mo, and ultimately alleviate the symptoms of molybdenum poisoning [50]. In this experiment, *P. picticaudata* with severe diarrhea were fed 100 mg of Cd·kg^−1^ in body weight once every 5 days; then, the diarrhea symptoms of *P. picticaudata* fed with CuSO_4_ were alleviated and treated, the hematological indices of the *P. picticaudata* improved, and the level of Mo in the blood was lowered to the normal level, whereas the diarrhea symptoms of *P. picticaudata* not supplied with CuSO_4_ were not improved and there was no significant change in relevant parameters in the body, indicating that CuSO_4_ can alleviate the symptoms of molybdenosis in *P. picticaudata*.

## 5. Conclusions

The high Mo content in the soil and forage caused a significant increase in the Mo content in the blood, leading to molybdenosis in the *P. picticaudata* in ranches of the animal rescue center. Molybdenosis caused Cu deficiency and severely reduced the antioxidant capacity of the *P. picticaudata*. The state of oxidative stress is likely to disrupt the function of the liver, kidneys and muscles, whereas CuSO_4_ supplementation can significantly alleviate the symptoms of molybdenosis by reducing the molybdenum content in the blood.

## Figures and Tables

**Table 1:** Main nutritional components of mixed forage in experimental areas (%).

Nutrient Components	Rescue Center	Healthy Ranches
DM	94.04 ± 2.51	94.58 ± 2.39
CP	16.67 ± 0.79	16.42 ± 0.87
EE	4.33 ± 0.38	4.39 ± 0.35
CF	19.69 ± 1.52	19.77 ± 1.47
CA	9.52 ± 0.76	9.68 ± 0.73
NFE	44.13 ± 1.15	45.07 ± 1.21
NDF	44.61 ± 0.29	44.77 ± 0.39
ADF	26.73 ± 1.63	26.91 ± 1.68

DM, dry matter; CP, crude protein; EE, ether extract; CF, coarse fiber; CA, crude ash; NFE, nitrogen-free extract; NDF, neutral detergent fiber; ADF, acid detergent fiber.

**Table 2:** The hematological levels in the *P. picticaudata*.

Parameters	Affected Animals	Healthy Animals
Hb (g·L^−1^)	81.6 ± 7.59 *	117 ± 7.60
RBC (10^12^ L^−1^)	6.54 ± 0.41	6.45 ± 0.53
PCV (%)	35.9 ± 3.67 *	51.3 ± 7.30
MCV (fL^−1^)	45.5 ± 3.16 *	53.9 ± 2.04
MCH (pg)	9.93 ± 1.44 *	14.73 ± 0.84
MCHC (%)	22.9 ± 1.70	22.1 ± 1.67
WBC (10^9^ L^−1^)	8.31 ± 0.59	8.03 ± 0.64

* Indicates significant difference at *p* < 0.01.

**Table 3:** The biochemical parameters in the *P. picticaudata*.

Parameters	Affected Animals	Healthy Animals
Cp (mg·L^−1^)	3.48 ± 11.69 *	6.82 ± 11.31 *
ALB (g·L^−1^)	20.1 ± 3.17 *	30.4 ± 2.17
ALP (U·L^−1^)	941 ± 96 *	635 ± 65
ALT (U·L^−1^)	45.1 ± 5.18	43.1 ± 3.24
AST (U·L^−1^)	127 ± 5.49 *	84.40 ± 12.1
CPK (U·L^−1^)	386 ± 39 *	250 ± 12
CR (µmoL·L^−1^)	64.5 ± 6.17	63.2 ± 3.46
Chol (mmol·L^−1^)	1.79 ± 0.12	1.72 ± 0.19
GLB (g·L^−1^)	14.9 ± 3.32 *	24.6 ± 4.26
LDH (U·L^−1^)	536 ± 31 *	420 ± 33
GSH-Px (U·mL^−1^)	134 ± 16 *	329 ± 20
SOD (U·mL^−1^)	35.4 ± 3.37 *	92.7 ± 8.85
CAT (U·mL^−1^)	2.96 ± 0.30 *	13.31 ± 1.17
T-AOC (U·mL^−1^)	2.10 ± 0.08 *	5.49 ± 0.31
MDA (nmol·L^−1^)	41.5 ± 2.68 *	14.2 ± 1.28
TP (g·L^−1^)	37.1 ± 4.94 *	54.3 ± 3.97

* Indicates significant difference at *p* < 0.01.

**Table 4:** Effect of supplying CuSO_4_ on the hematological levels in the *P. picticaudata*.

Parameters	Treatment Group	Control Group
Hb (g·L^−1^)	99.7 ± 8.61 *	83.2 ± 7.47
PCV (%)	46.9 ± 3.52 *	34.5 ± 3.38
MCV (fL^−1^)	48.5 ± 4.03	44.2 ± 3.21
MCH (pg)	12.9 ± 1.39 *	9.7 ± 1.19

* Indicates significant difference at *p* < 0.01.

**Table 5:** Effect of supplying CuSO_4_ on the biochemical parameters in the *P. picticaudata*.

Parameters	Treatment Group	Control Group
Cp (mg·L^−1^)	5.21 ± 1.69 *	3.72 ± 1.51
ALB (g·L^−1^)	28.2 ± 3.02 *	20.3 ± 2.21
ALP (U·L^−1^)	679 ± 71 *	933 ± 89
AST (U·L^−1^)	103 ± 6.41 *	128 ± 5.31
CPK (U·L^−1^)	339 ± 28 *	388 ± 24
GLB (g·L^−1^)	20.3 ± 3.17 *	14.6 ± 3.14
LDH (U·L^−1^)	470 ± 27 *	539 ± 27
GSH-Px (U·mL^−1^)	239 ± 28 *	132 ± 17
SOD (U·mL^−1^)	60.1 ± 5.39 *	36.2 ± 3.29
CAT (U·mL^−1^)	7.66 ± 1.31 *	2.86 ± 0.29
T-AOC (U·mL^−1^)	4.11 ± 0.12 *	2.17 ± 0.11
MDA (nmol·L^−1^)	20.4 ± 3.18 *	42.3 ± 3.18
TP (g·L^−1^)	46.1 ± 5.64 *	36.9 ± 4.33

* Indicates significant difference at *p* < 0.01.

## Data Availability

Data are provided upon request due to privacy restrictions. The data provided in this study are available upon request from the corresponding author. As this paper is part of a series of studies, the data are not publicly available.
